# Comparative analysis of isoxazoline activity on human and canine GABA receptors expressed in *Xenopus* oocytes

**DOI:** 10.1186/s13071-025-06847-3

**Published:** 2025-06-06

**Authors:** Heinz Sager, Anouk Sarr, Emmanuelle Kuntz, Lucien Rufener

**Affiliations:** 1 Elanco Animal Health, Mattenstrasse 24a, 4058 Basel, Switzerland; 2 Invenesis Sàrl, Rue de Neuchâtel 15A, 2072 St-Blaise, Switzerland

**Keywords:** Isoxazoline, Lotilaner, Fluralaner, Sarolaner, Afoxolaner, GABAR, *Xenopus* oocytes

## Abstract

**Background:**

Isoxazolines, including sarolaner, lotilaner, afoxolaner and fluralaner, are a class of ectoparasiticides that target gamma-aminobutyric acid receptors (GABARs) in insects and acari. However, their potential action on mammalian GABARs has not been extensively compared.

**Methods:**

This study investigated the inhibitory effects of these isoxazolines on human and canine GABARs expressed in *Xenopus* oocytes. Eleven functional GABAR subunit combinations from human and canine isoforms were successfully cloned and expressed. Two-electrode voltage-clamp measurements were performed to determine the inhibitory effects of the isoxazolines.

**Results:**

Sarolaner, afoxolaner and fluralaner exhibited partial to high inhibition of human and canine GABARs, with fluralaner showing the lowest half-maximal inhibitory concentration (IC_50_) values (1.9–13 µM). In contrast, lotilaner had little or no inhibitory effect, with IC_50_ values > 30 µM for both human and canine GABARs.

**Conclusions:**

While neurological adverse events have been reported in dogs, particularly in breeds with the multidrug resistance 1 (MDR1) gene mutation, this study suggests that direct inhibition of canine GABARs may not be the primary cause. However, the interpretation of these results represents a challenge, and a direct correlation with documented cases of adverse events remains difficult. Further research is needed to understand the exposure of mammalian GABARs to isoxazolines and the analog-specific safety risks associated with the observed in vitro receptor activities.

**Graphical Abstract:**

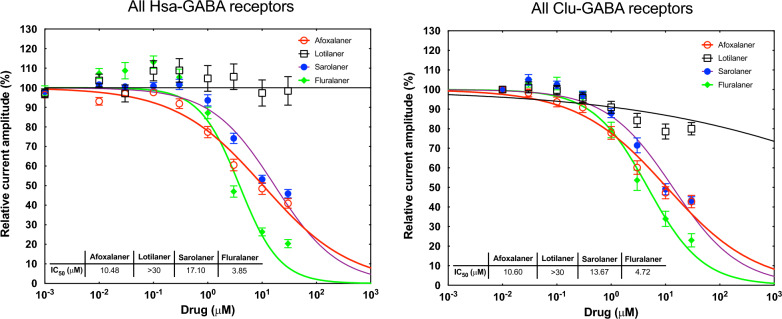

**Supplementary Information:**

The online version contains supplementary material available at 10.1186/s13071-025-06847-3.

## Background

Building on over two decades of research and development, isoxazolines have emerged as a highly effective class of ectoparasiticides [1–3]. Isoxazoline compounds, including afoxolaner, fluralaner, sarolaner and lotilaner, target ligand-gated chloride channels (LGCCs) in insects and acari, specifically those receptors regulated by glutamate and γ-aminobutyric acid (GABA). This mechanism delivers potent insecticidal and acaricidal activities, with limited inhibition of rodent GABA receptors (GABARs) [4].

Ionotropic GABARs are members of the large pentameric LGCCs (previously referred to as "Cys-loop" receptors) superfamily of evolutionarily related and structurally similar ligand-gated ion channels, including nicotinic acetylcholine receptors, glycine receptors and the 5-HT_3_ receptor [5]. GABARs are ligand-gated ion channels composed of five protein subunits that form a chloride-permeable pore and play a critical role in transmembrane signaling in neurons and muscle cells [6]. The endogenous ligand is GABA, a major inhibitory neurotransmitter in the central nervous system [7]. Upon activation, the GABAR selectively conducts chloride anions (Cl^−^) through its pore. The receptor sits in the membrane of its neuron, typically localized in the post-synaptic neuronal membrane at a synapse. Each subunit of the GABAR features a large extracellular region at its N-terminus that is responsible for binding to the neurotransmitter GABA. The subunit then spans the cell membrane four times (via transmembrane domains M1-M4), with the M2 region forming the lining of the central chloride channel [8–10].

There are two pharmacologically distinct categories of ionotropic GABARs found in vertebrates: bicuculline-sensitive GABA_A_ receptors, which are allosterically modulated by benzodiazepines and barbiturates, and bicuculline-insensitive GABA_C_ receptors, which are insensitive to the majority of modulators of GABA_A_ receptors [11]. Unlike vertebrate GABARs, which fall into the GABA_A_ and GABA_C_ categories, arthropod ionotropic GABARs (RDL GABARs) defy easy categorization. Most insect genomes contain a single ionotropic GABAR subunit gene, *Rdl,* which forms homomeric GABA-gated chloride channels [12]. Although insensitive to the GABA_A_ antagonist bicuculline, they surprisingly display weak allosteric modulation by GABA_A_ modulators, such as benzodiazepines and barbiturates [13].

In vertebrates, at least 21 known subunit types of GABA_A_ receptors have been identified: α1–6, β1–4, γ1–4, δ, ρ1–3, θ and π. Although these receptors exhibit extensive subunit diversity, combinations of two α subunits, two β subunits and one γ subunit are the most common [14]. The α1β2γ2 configuration is the most prevalent in the central nervous system, followed by α2β3γ2 and α3β3γ2. Receptors incorporating α4, α5, α6, β1, γ1, γ3, δ, π or θ subunits are less common, representing a smaller proportion of the total GABA_A_ receptors [14]. Unlike synaptic GABA_A_ receptors, those assembled from αβ or αβδ subunits are predominantly located extrasynaptically, allowing them to generate tonic inhibition [15]. The affinity and efficacy of barbiturates depend on the composition of the subunit, but the α subunit seems to be more important than the β subunit [16, 17].

The effectiveness of isoxazolines is attributed to their function as noncompetitive antagonists of GABARs. These compounds exhibit significantly higher selectivity for GABARs in target parasites, such as fleas and ticks, compared to those found in vertebrates, including humans [3, 18]. By binding to arthropod GABA-gated chloride channels (GABACls) in both nerve and muscle cells, isoxazolines effectively inhibit neuronal signal transmission, resulting in paralysis and death of the targeted parasites. This mechanism of action is further corroborated by the documented safety profile of registered isoxazolines for canine and feline administration, indicating a high degree of selectivity for arthropods versus mammalian GABAR. Reports of neurological adverse events (AEs), including seizures, tremors and ataxia, as well as gastrointestinal symptoms, including vomiting and diarrhea, in dogs treated with isoxazoline parasiticides have raised concerns regarding the specificity of these compounds for insect and acarid GABARs versus mammalian GABARs. Palmieri and colleagues examined the use and safety of isoxazoline parasiticides administered to dogs and documented severe AEs, predominantly serious neurologic effects and fatalities in canines [2]. Limited information is available on AEs in humans upon exposure to isoxazolines. However, the question of safety risks has become more pertinent due to the recent use of lotilaner for the treatment of blepharitis of *Demodex* spp. origin [19]. Further utilization of isoxazolines in human medicine for the control of vector-borne diseases has been proposed [20].

The objective of this study was to assess the parasite-specificity of isoxazolines by providing evidence for specific activities of afoxolaner, fluralaner, sarolaner and lotilaner on human and canine GABA ion channels expressed in *Xenopus* oocytes. This study provides a framework for exploring the potential correlation between AEs and GABAR specificity.

## Methods

### Cloning of human and canine GABAA cDNA sequences

 Complementary DNA (cDNA) encoding for the human GABA_A_ proteins α1 (NM_001127645.2), α2 (NM_001377146.1), α3 (KJ620007.1), α4 (NM_000809.4), α5 (NM_000810.4), α6 (NM_000811.3), β2 (XM_054352312.1), β3 (M82919.1), γ2 (NM_000816.3) and δ (NM_000815.5) were ordered from Genewiz (www.genewiz.com; Leipzig, Germany); for each gene, GenBank accession numbers are provided in parentheses. When necessary, silent mutations were introduced to break NheI or XhoI internal restriction sites. The corresponding open reading frames (ORF) were cloned into the pT7-TS transcription vector (introducing *X. laevis* β-globin untranslated cDNA to the 5’ and 3’ ends of the gene), following a procedure described in detail in a previous publication [21]. The same process was used to obtain cDNA encoding canine GABA_A_ proteins α4 (XM_025435560.1), α6 (XM_022417729.1) and δ (XM_005620414.2). Gene α2 (XM_025435664.1), α3 (XM_549343.5), α5 (XM_545805.6) and β3 (XM_025437408.1) were obtained by PCR; GenBank reference accession numbers are provided in parentheses. RNA extraction, cDNA synthesis and PCR amplification were performed as described previously [21]. Total RNA was extracted from a piece of dog brain (Beagle breed; *Canis lupus familiaris*) from which 1 µg of total RNA (DNase-treated) was reverse-transcribed to cDNA using a (dT)30 primer and SuperScript III Reverse Transcriptase (Invitrogen, Thermo Fisher Scientific, Waltham, MA, USA). Gene-specific primers (Additional file 1: Table S1) were designed using Primer 3 software (http://frodo.wi.mit.edu/). The following primers were used to amplify the full-length coding sequences based on published sequences: primers BglII_Clu-GABA-a2_F and XhoI_Clu-GABA-a2_R, for Clu-GABAa2; primers NheI_Clu-GABA-a3_F and XhoI_Clu-GABA-a3_R, for Clu-GABAa3; primers NheI_Clu-GABA-a5_F and XhoI_Clu-GABA-a5_R, for Clu-GABA-a5; primers NheI_Clu-GABA-b3_F and XhoI_Clu-GABA-b3_R, for Clu-GABA-b3 (Additional file 1: Table S1). Gene-specific PCR to obtain full-length Clu-GABA subunits from *Canis lupus familiaris* cDNA was performed using a Phusion polymerase (New England Biolabs, Ipswich, MA, USA) and the primer pairs described in Additional file 1: Table S1. The cycling conditions were: 98 °C for 30 s; then 38 cycles of 98 °C for 10 s, 58 °C for 30 s and 72 °C for 90 s; followed by 72 °C for 5 min. The dog subunits α1 (XM_546261.5), β2 (XM_014113040.1) and *γ*2 (XM_546259.5) had been previously amplified and described in a study by Rufener et al. [22].

### Expression of GABACls in *Xenopus laevis* oocytes

Defoliculated* X. laevis* oocytes ordered from Ecocyte Bioscience (Dortmund, Germany) were microinjected with 15–25 nl of cRNA solution (5–50 ng/μl) per oocyte using an automated injector (Roboinject; Multi Channel Systems MCS GmbH, Reutlingen, Germany) and then incubated at 18 °C. All recordings were made 1–5 days post-cRNA injection and were performed at 18 ± 4 °C. Cells were superfused with OR2 medium (NaCl 88 mM, KCl 2.5 mM, HEPES 5 mM, CaCl_2_•2H_2_O 1.8 mM, MgCl_2_•7H_2_O 1.0 mM, pH 7.4). To ensure that γ2 subunits were integrated into the heteropentamers, we injected fivefold more γ2 than the other subunit cRNAs. Diazepam is a positive allosteric modulator of GABAR only if γ2 subunits are present [23]. Therefore, we used this method to confirm the presence of human and dog GABA_A_ receptor containing the γ2 subunit. For all receptor subtypes expressed in this study, a strong positive modulation (> 200%) with diazepam was observed, demonstrating the incorporation of the γ2 subunit within the heteromeric complex (data not shown).

Eleven functional GABAR subunit combinations, comprising six human (*Homo sapiens sapiens* [Hsa]) subunit combinations (α1β2γ2, α2β2γ2, α2β3γ2, α3β2γ2, α5β2γ2, α6β2γ2) and five canine (*C. lupus familiaris* [Clu]) isoforms (α1β2γ2, α2β2γ2, α2β3γ2, α3β2γ2, α5β2γ2), were successfully cloned and expressed in *X. laevis* oocytes. The human α4β2δ, canine α4β2δ and α6β2γ2 subunit combinations did not express in *X. laevis* oocytes.

### Two-electrode voltage-clamp measurements using the HiClamp

Voltage-clamp measurements were performed as described by Rufener et al. [22]. Oocytes were impaled with two electrodes filled with 3 M KCl, and their membrane potentials were maintained at − 80 mV throughout the experiment. Cells showing a significant current induced by a brief GABA pulse (concentration adjusted based on the receptor subtype tested) were used to measure dose responses. Recordings were performed using an automated process equipped with standard two-electrode voltage-clamp configuration (HiClamp system; Multi Channel Systems MCS GmbH). Data were filtered at 10 Hz, captured at 100 Hz and analyzed using proprietary data acquisition and analysis software in MATLAB (Mathworks Inc., Natick, MA, USA). Additional analyses were performed using Excel (Microsoft, Redmond, WA, USA). To determine the respective GABA half maximal (median) effective concentration (EC_50_) and Hill Slope (slope factor), we used a protocol with successive growing concentration exposures for 10 s of GABA. Since the oocytes were challenged several times with GABA, a sufficient resting time (60 s) was allowed between treatments for the receptor to recover from desensitization. Graphing the maximum inward currents against the log of agonist concentration produces typical concentration-activation and concentration-inhibition curves, which can be accurately described using individual Hill equations. The concentration-activation curves were fitted using the following equation:

$$Y=\frac{100}{1+{10}^{H(logEC50-X)}}$$

where *Y* is the normalized response, logEC_50_ is the logarithm of the concentration of agonist eliciting half-maximal current amplitude, *X* is the log of the dose or concentration, and *H* is the slope factor or Hill slope. The same equation was used for the concentration-inhibition curves but logEC_50_ was replaced by logIC_50_ (half-maximal inhibitory concentration).

To assess the antagonistic properties of the four test compounds, oocytes transfected with the previously described human and canine GABA subtypes were sequentially pre-exposed for 60 s to the tested compounds at 0, 30, 100 and 300 nM and 1, 3, 10 and 30 μM. After each exposure, the compounds were co-applied for 10 s with GABA at concentrations near the EC_10_ (determined separately for each receptor subtype; see Table 1). The agonist and the drug were then washed off for 15 s and the oocyte was exposed again to the same drug concentration for 25 s before increasing to the next concentration. To establish a baseline response, GABA was initially applied 3–5 times for 10 s every 1.5 min at the beginning of the experiment.

**Table 1:** Receptor response profiles to their natural agonist

Receptors	Agonist	GABA concentration (near EC_10_)^a^	EC_50_ (μM)^b^	95% CI^b^	n_H_c	95% CI^c^	*N*^d^
Hsa-GABA α1β2γ2	GABA	1 µM	8.2	6.5–10.3	1.7	1.1–2.2	2
Hsa-GABA α2β2γ2	GABA	3 µM	17	14.1–20.6	1.4	1.1–1.7	3
Hsa-GABA α2β3γ2,	GABA	1 µM	10.6	9.1–12.3	1.2	1.0–1.4	5
Hsa-GABA α3β2γ2	GABA	1 µM	12.4	9.7–15.8	1.2	0.9–1.4	2
Hsa-GABA α5β2γ2	GABA	0.3 µM	3.2	2.6–4.0	1.1	0.9–1.3	2
Hsa-GABA α6β2γ2	GABA	0.3 µM	0.6	0.5–0.8	1.1	0.9–1.3	4
Hsa-GABA α4β2δ	GABA	NA	NA	NA	NA	NA	NA
Clu-GABA α1β2γ2	GABA	1 µM	10.0	7.7–13.0	1.0	0.8–1.2	4
Clu-GABA α2β2γ2	GABA	5 µM	42	38.2–46.1	1.3	1.2–1.4	4
Clu-GABA α2β3γ2	GABA	1 µM	8.6	7.9–9.4	1.6	1.4–1.7	3
Clu-GABA α3β2γ2	GABA	0.5 µM	40.4	31.5–51.7	0.8	0.7–0.9	
Clu-GABA α5β2γ2	GABA	1 µM	8.1	7.4–8.9	1.1	1.0–1.2	4
Clu-GABA α6β2γ2	GABA	NA	NA	NA	NA	NA	NA
Clu-GABA α4β2δ	GABA	NA	NA	NA	NA	NA	NA

*CI* Confidence interval, *Clu* *Canis lupus familiaris*, *EC*_*10*_ 10% effect concentration,* GABA* gamma-aminobutyric acid,* Hsa* *Homo sapiens sapiens*,* NA* not available

^a^The GABA concentrations near the EC_10_ are listed for each receptor

^a^Half-maximal effective concentration (EC_50_) ± 95% CI

^c^Hill coefficient ± 95% CI

^d^Number of oocytes evaluated

## Results

### Functional characterization of Hsa- and Clu-GABA receptors

Upon challenge with GABA (10-s-long applications), all GABAR subtypes demonstrated a characteristic fast opening (at high doses) with a slow desensitization phase (Additional file 2: Figure S1). For Hsa-GABAR, the EC_50_ for GABA ranged from 0.6 μM (95% confidence interval [CI] 0.5–0.8 μM; Hsa-GABA α6β2γ2, *n* = 4) to 17.0 (95% CI 14.1–20.6 μM; Hsa-GABA α2β2γ2, *n* = 3). Hill coefficients were higher than 1 for the six receptors. For Clu-GABAR, the EC_50_ for GABA ranged from 8.1 μM (95% CI 7.4–8.9 μM; Clu-GABA α5β2γ2, *n* = 4) to 42.0 (95% CI 38.2–46.1 μM; Clu-GABA α2β2γ2, *n* = 4). The Hill coefficients varied from 0.8 to 1.6 for the five receptors; the receptor response profiles are listed in Table 1.

### Isoxazolines are weak antagonists of mammalian GABAR

**Fig. 1:**
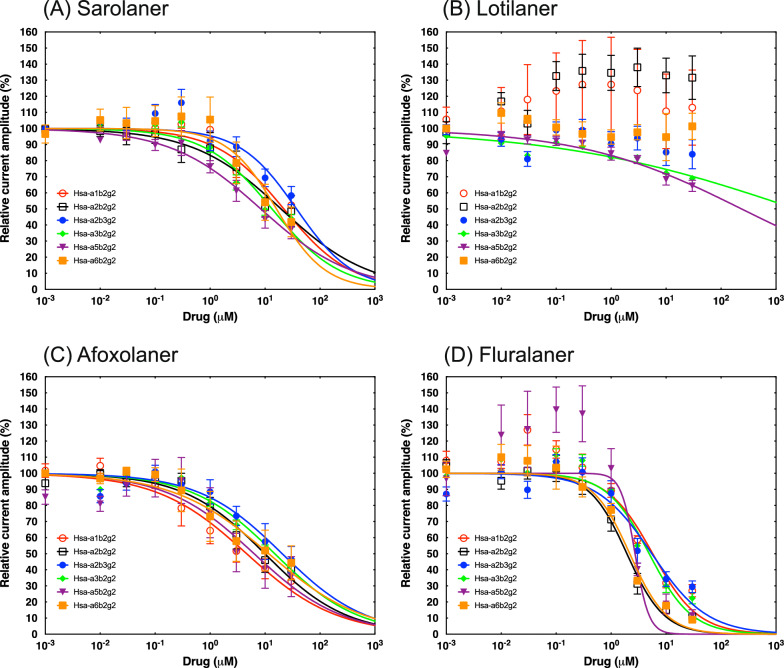
Averaged inhibitory dose–response curves for human GABA receptor subtypes. Individual curves were standardized to the fitted maximal current amplitude and subsequently averaged. The mean ± standard error of the mean (SEM) of experiments carried out with ≥ 3 oocytes is shown. GABA, Gamma-aminobutyric acid

**Fig. 2:**
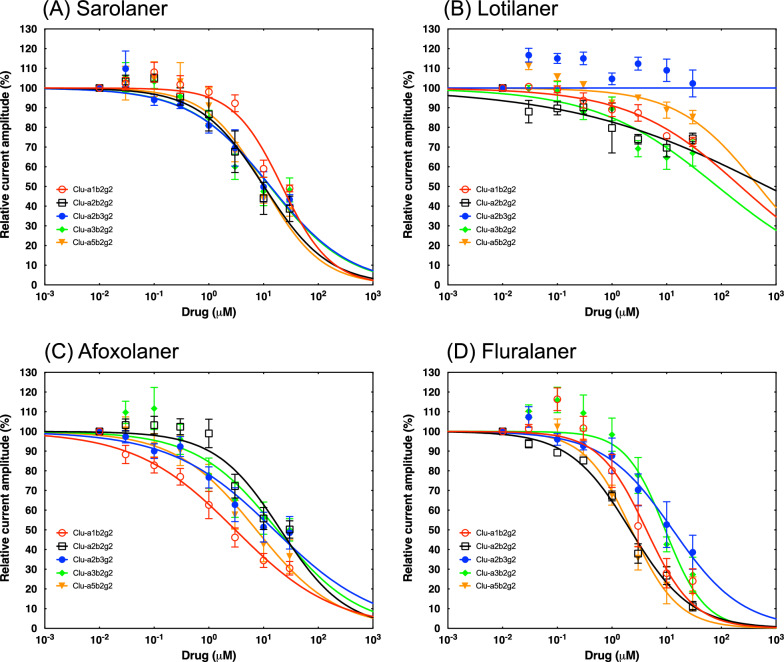
Averaged inhibitory dose–response curves for dog GABA receptor subtypes. Individual curves were standardized to the fitted maximal current amplitude and subsequently averaged. The mean ± standard error of the mean (SEM) of experiments carried out with ≥ 3 oocytes is shown. GABA, Gamma-aminobutyric acid

When sarolaner, afoxolaner and fluralaner were applied to Hsa-GABARs, an average inhibitory effect of 53%, 59% and 82% GABA current inhibition at 30 μM, respectively, was observed; the average 30 μM inhibition on Clu-GABARs was 57%, 57% and 76%, respectively (Fig. 1a, c, d, and Fig. 2a, c, d). Lotilaner exposure to Hsa- and Clu-GABARs had little or no inhibitory effect, with 13% (Hsa-GABARs) and 20% (Clu-GABARs) average GABA current inhibition at 30 μM (Figs. 1b, 2b). The concentration that resulted in IC_50_ was determined for all compounds and subunit combinations (Table 2). Fluralaner showed the lowest IC_50_ values on both human and canine receptors, ranging from 1.9 to 5.7 µM for the Hsa GABAR subunit-combinations and from 2.2 to 13 µM for the Clu GABAR subunit combinations. Sarolaner and afoxolaner generally had higher IC_50_ values for both Hsa- and Clu-GABARs than fluralaner, starting at 3 µM for afoxolaner for Clu-GABA α1β2γ2 and 8.4 µM for sarolaner for Hsa-GABA α5β2γ2 (Table 2). Afoxolaner IC_50_ for human GABAR ranged from 4.6 to 20.5 µM, and for canine GABAR, from 3 to 20.6 µM. Sarolaner IC_50_ for human GABAR ranged from 8.4 to > 30 µM, and for canine GABAR, from 10.2 to 21.8 µM. In contrast, all IC_50_ values of lotilaner were above 30 µM for both Hsa- and Clu-GABARs (Table 2). Representative current traces for all receptor subtypes exposed to the four compounds are illustrated in Additional file 3: Figures S2–S5.

**Table 2:** Response profiles of receptors to the four isoxazolines

Receptors	Sarolaner			Lotilaner			Afoxolaner			Fluralaner		
	IC_50_^a^	95% CI^a^	*N*^b^	IC_50_^a^	95% CI^a^	*N*^b^	IC_50_^a^	95% CI^a^	*N*^b^	IC_50_^a^	Fluralaner 95% CI^a^	*N*^b^
Hsa-GABA α1β2γ2	21.9	15.7–33.7	4	> 30	NA	3	4.6	2.8– to 8.0	3	**5.7**	3.8– to 8.8	6
Hsa-GABA α2β2γ2	19.5	10.5–52.2	4	> 30	NA	5	9.9	6.4– to 16.8	5	**1.9**	1.5– to 2.5	3
Hsa-GABA α2β3γ2	> 30	NA	3	> 30	NA	4	20.5	11.9– to 49.3	4	**5.5**	3.8– to 8.4	7
Hsa-GABA α3β2γ2	13.4	10.0–18.8	4	> 30	NA	3	15.0	11.4– to 20.7	3	**4.9**	3.7– to 6.7	3
Hsa-GABA α5β2γ2	8.4	6.6–10.9	4	> 30	NA	3	6.3	3.7– to 12.4	4	**2.7**	1.8– to 5.3	4
Hsa-GABA α6β2γ2	16.1	12.5–21.5	8	> 30	NA	3	11.7	7.6– to 19.7	5	**2.2**	1.8– to 2.7	4
Clu-GABA α1β2γ2	21.8	16.2–32.3	4	> 30	NA	3	3.0	2.1– to 4.5	6	**4.3**	2.8– to 6.8	3
Clu-GABA α2β2γ2	10.2	6.65–17.1	3	> 30	NA	6	20.6	13.7– to 35.4	5	**2.2**	1.8– to 2.6	4
Clu-GABA α2β3γ2	13.4	8.45–24.2	5	> 30	NA	3	15.7	8.8– to 35.1	9	**13**	8.1– to 23.6	3
Clu-GABA α3β2γ2	13.1	7.88–25.0	4	> 30	NA	5	17.9	8.7– to 53.7	3	**9.4**	6.3– to 14.6	3
Clu-GABA α5β2γ2	10.2	7.71–13.9	4	> 30	NA	4	7.4	5.9– to 9.5	6	**2.3**	1.7– to 3.0	3

*CI* Confidence interval, *Clu* *Canis lupus familiaris*, *GABA* gamma-aminobutyric acid,* Hsa* *Homo sapiens sapiens*,* NA* not available

^a^Half-maximal inhibitory concentration in μM (IC_50_ ± 95% CI)

^b^Number of oocytes evaluated

## Discussion

This is the first extensive comparative study of four isoxazoline analogs on mammalian GABAR. In the current study, lotilaner did not show any significant inhibition of any of the human and canine GABARs tested up to 30 μM. Sarolaner showed partial inhibition of human GABARs, with the subtype α5β2γ2 being the most sensitive. Afoxolaner showed partial inhibition of human GABAR subtypes α1β2γ2 and α5β2γ2, with IC_50_ values < 10 μM. Fluralaner showed high inhibition of human GABARs, with IC_50_ values < 10 μM and > 90% current inhibition at 30 μM on α2β2γ2, α5β2γ2 and α6β2γ2.

Isoxazolines are generally considered to be safe for use in vertebrates. Although most dogs and cats tolerate isoxazolines without neurological issues, some cases of muscle tremors, ataxia and seizures have been reported in association with the use of isoxazolines. Therefore, the possibility of pharmacodynamic effects on the vertebrate central nervous system remains [24]. In a recent study, brain penetration of fluralaner was observed in an *mdr1* mutant mouse model, indicating that fluralaner entry into the brain is prevented by multidrug resistance 1 (MDR1)-mediated drug efflux [25]. Hence, dogs with MDR1 mutations, such as Collies, Australian Shepherds, Shetland Sheepdogs, Longhaired Whippets, White Swiss Shepherds and some other breeds may have a greater exposure of GABAR to isoxazolines than other dog breeds. Studies with lotilaner in MDR1 mutant dog breeds did not reveal neurological symptoms and have confirmed the high safety profiles [26]. Of note, GABARs are also present in various peripheral tissues, including pancreas, liver, kidney, gastrointestinal tract, trachea, immune cells and vessels but may be less affected by MDR1-mediated efflux.

In the in vitro assays, concentrations of isoxazolines from 10^-3^ to 30 µM were tested on the expressed GABA-channels. The maximum in vivo concentrations in dog plasma ranged between 1.9 µM (sarolaner) and 6.7 µM (lotilaner) after oral treatment at the minimum recommended dose. It can be assumed that the concentration in the brain will not exceed these values. Fluralaner administered at a dose of 25 mg/kg body weight may reach a maximum plasma concentration of 3400 ng/ml, which corresponds to 6.1 µM. In this case, an inhibitory effect may occur in at least three of the five examined canine GABARs. Although the hypothetical exposure of humans is expected to be much lower, even in case of accidental uptake, the high activity of fluralaner on the human receptor-combinations with an IC_50_ ranging between 1.9 and 5.7 µM may still pose a risk. Afoxolaner had one canine and three human GABA subunit combinations that presented IC_50_ values in the range of 3–9.9 µM, which is close to its identified C_max_ (peak concentration in the bloodstream) of 4.7 µM after oral dosing at 2.5 mg/kg. Sarolaner had lower plasma concentrations, and only the human subunit combination α5β2γ2 showed an IC_50_ that was positioned in the order of magnitude of the C_max_ of 1.9 µM measured in dog plasma after treatment at a dose of 2 mg/kg body weight. All IC_50_ values for lotilaner were above 30 µM. It can therefore be assumed that despite the relatively high maximum plasma concentrations of 6.7 µM, this analog poses the least risk for the inhibition of human and canine GABA ion channels [27]. The safety survey by Palmieri et al. [2] was based mostly on data collected from 2013 to 2017. Therefore, the focus of that study was on fluralaner, afoxolaner and sarolaner, whereas lotilaner was only mentioned in the European Medicines Agency (EMA) AE report with an extended collection period from 2013 to 2019. Therefore, it is not possible to identify a correlation between the observed number of AE cases and our findings of human and canine GABAR sensitivity to different isoxazoline analogs. Further research is needed to better understand the exposure of mammalian GABAR to isoxazolines and the analog-specific safety risks associated with receptor activities observed in vitro.

## Conclusions

This study identified analog-specific inhibitory activities of isoxazolines on canine and human GABAR in vitro. The interpretation of these results represents a challenge, and a direct correlation with documented AE cases remains difficult. Many parameters, such as maximum plasma concentrations, terminal half-life and the passage of the blood–brain barrier, define the in vivo exposure of receptors in the central nervous system. We focused on the targeted GABA_A_ receptors and the most frequent and relevant receptor subunit compositions. This represents only a small portion of the potential interactions that may occur in vivo. Nevertheless, there are clear differences in the inhibitory activities of isoxazoline analogs on various GABAR subunit compositions. Lotilaner was the only of the four tested molecules that were inactive against human and canine GABAR up to a concentration of 30 µM.

## Supplementary Information

Additional file 1: Figure S1. Current traces from a cumulative exposure to increasing dosage of GABA (in μM) on 6 and 5 different* Homo sapiens sapiens* and *Canis lupus familiaris* GABA receptor subtypes, respectively.

Additional file 2: Figure S2. Current traces from a co-application of GABA and increasing dosage of sarolaner (in μM) on 6 and 5 different* Homo sapiens sapiens* and* Canis lupus familiaris* GABA receptor subtypes, respectively.

Additional file 3: Figure S3. Current traces from a co-application of GABA and increasing dosage of lotilaner (in μM) on 6 and 5 different* Homo sapiens sapiens* and* Canis lupus familiaris* GABA receptor subtypes, respectively.

Additional file 4: Figure S4. Current traces from a co-application of GABA and increasing dosage of afoxolaner (in μM) on 6 and 5 different* Homo sapiens sapiens* and* Canis lupus familiaris* GABA receptor subtypes, respectively.

Additional file 5: Figure S5. Current traces from a co-application of GABA and increasing dosage of fluralaner (in μM) on 6 and 5 different* Homo sapiens sapiens* and* Canis lupus familiaris* GABA receptor subtypes, respectively.

Additional file 6: Table S1. Primers for full-length ORF amplification.

## Data Availability

No datasets were generated or analysed during the current study.
