# Supplementary material for: Comparative analysis of isoxazoline activity on human and canine GABA receptors expressed in Xenopus oocytes
Source: Parasit Vectors. 2025 Jun 6;18:213. doi: 10.1186/s13071-025-06847-3 (PMC12144822; doi:10.1186/s13071-025-06847-3)
Supplement: Supplementary file 2 — Additional file 2: Figure S2. Current traces from a co-application of GABA and increasing dosage of sarolaner (in μM) on 6 and 5 different Homo sapiens sapiens and Canis lupus familiaris GABA receptor subtypes, respectively. [file 13071_2025_6847_MOESM2_ESM.pdf]

Sarolaner

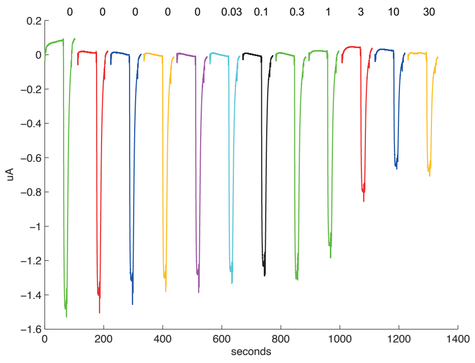

Hsa-GABA α1β2γ2

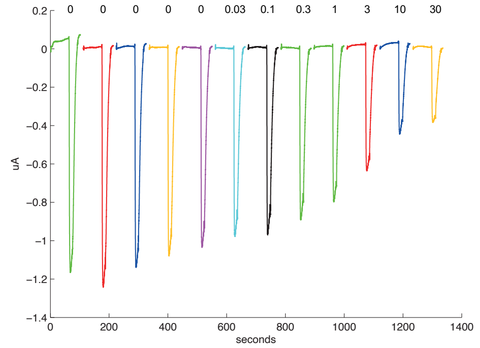

Hsa-GABA α2β2γ2

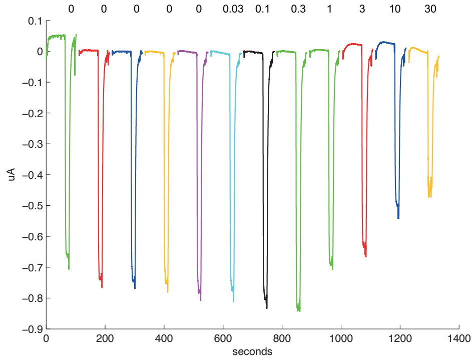

Hsa-GABA α2β3γ2

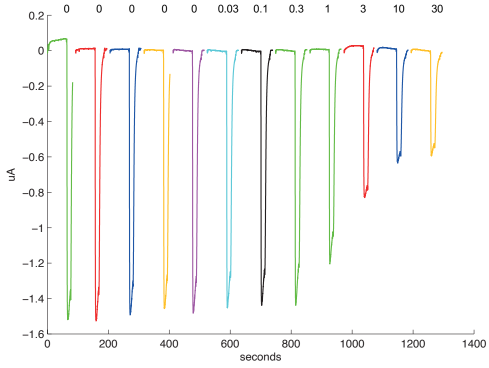

Hsa-GABA α3β2γ2

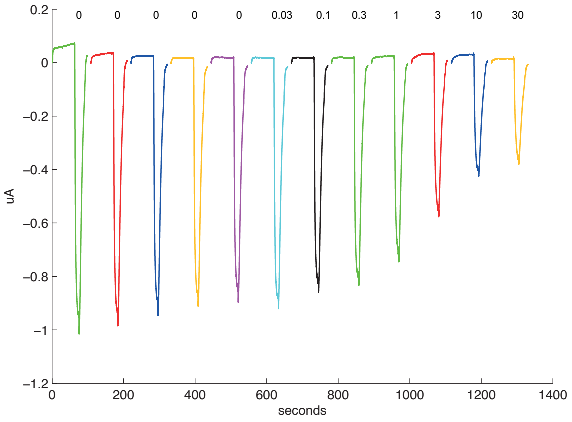

Hsa-GABA α5β2γ2

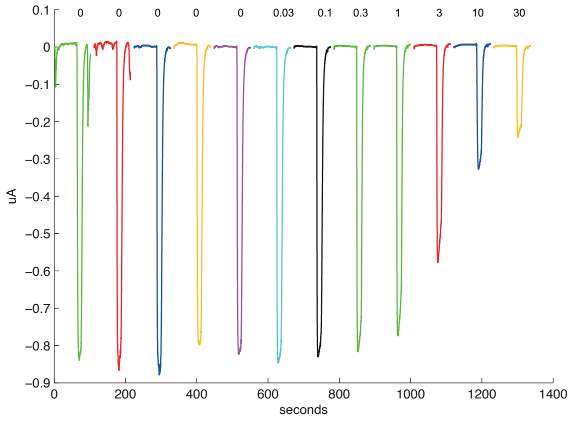

Hsa-GABA α6β2γ2

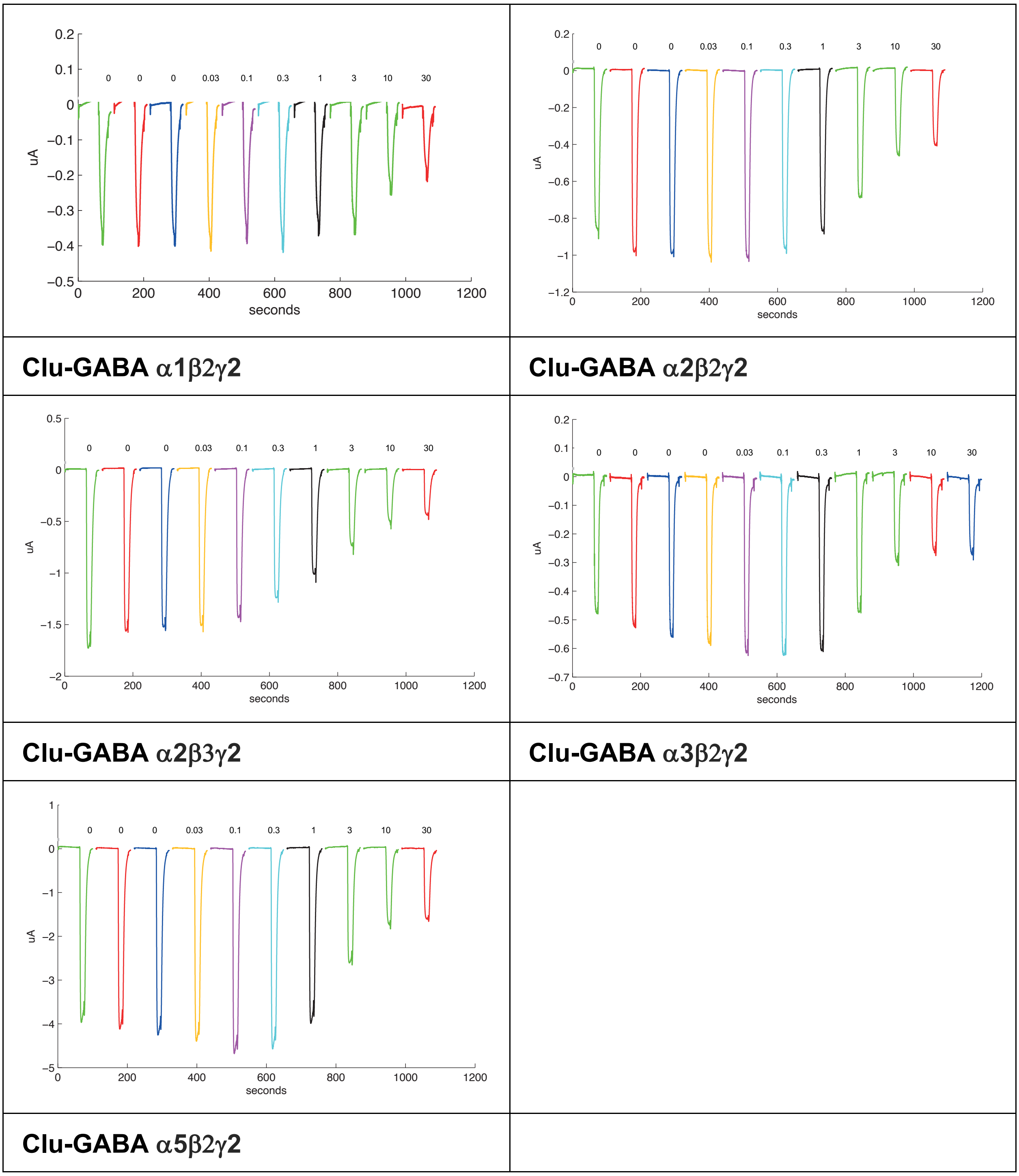

Supplementary Figure 2. Current traces from a co-application of GABA and increasing dosage of the compound (in μM) form respectively six and five different *Homo sapiens sapiens* and *Canis lupus familiaris* GABA receptor subtypes.
