# Supplementary material for: Comparative analysis of isoxazoline activity on human and canine GABA receptors expressed in Xenopus oocytes
Source: Parasit Vectors. 2025 Jun 6;18:213. doi: 10.1186/s13071-025-06847-3 (PMC12144822; doi:10.1186/s13071-025-06847-3)
Supplement: Supplementary file 3 — Additional file 3: Figure S3. Current traces from a co-application of GABA and increasing dosage of lotilaner (in μM) on 6 and 5 different Homo sapiens sapiens and Canis lupus familiaris GABA receptor subtypes, respectively. [file 13071_2025_6847_MOESM3_ESM.pdf]

Lotilaner

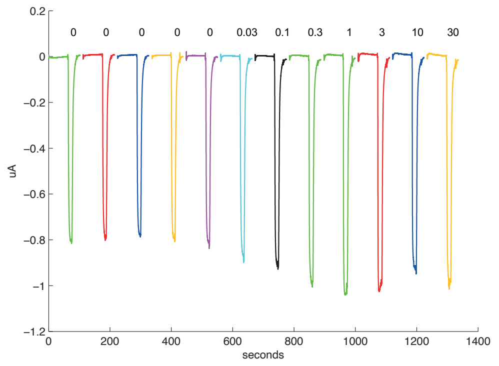	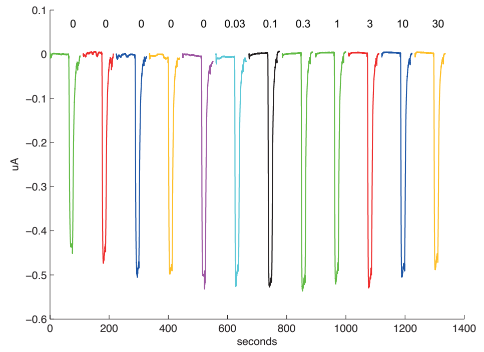
**Hsa-GABA α1β2γ2**	**Hsa-GABA α2β2γ2**
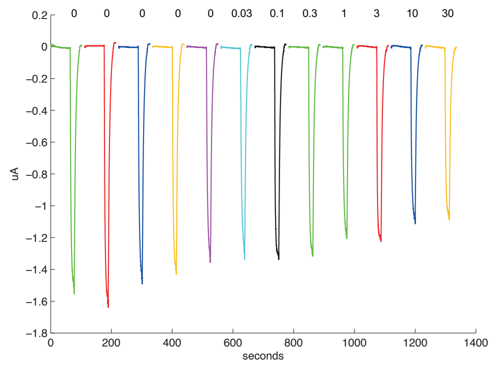	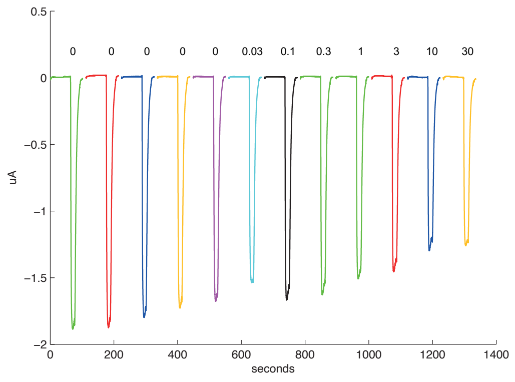
**Hsa-GABA α2β3γ2**	**Hsa-GABA α3β2γ2**
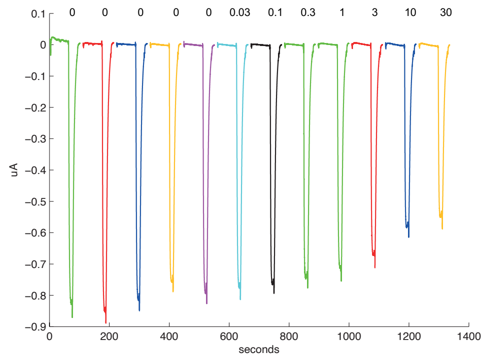	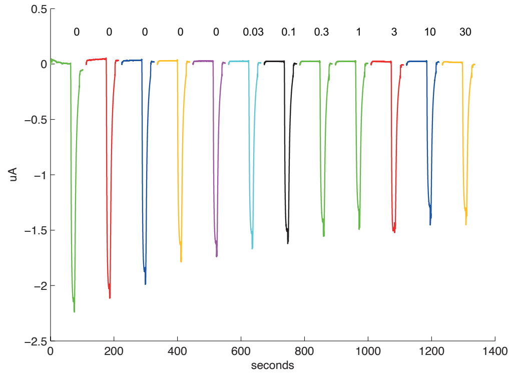
**Hsa-GABA α5β2γ2**	**Hsa-GABA α6β2γ2**

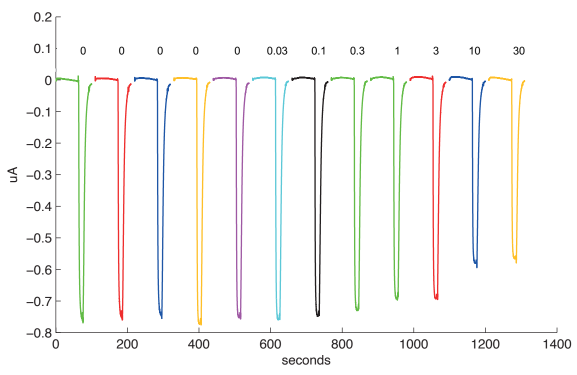

Clu-GABA α1β2γ2

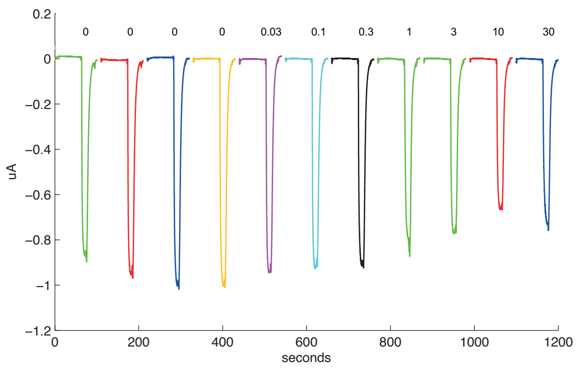

Clu-GABA α2β2γ2

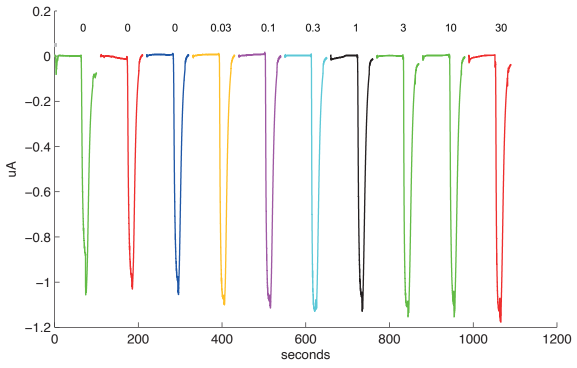

Clu-GABA α2β3γ2

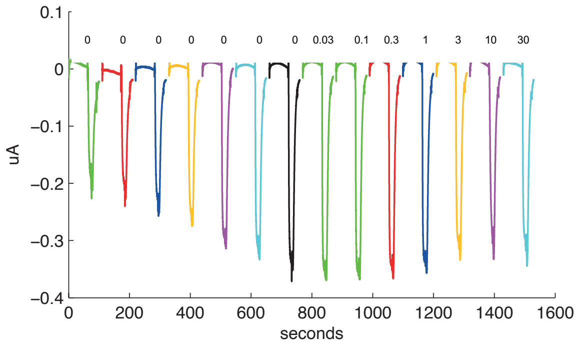

Clu-GABA α3β2γ2

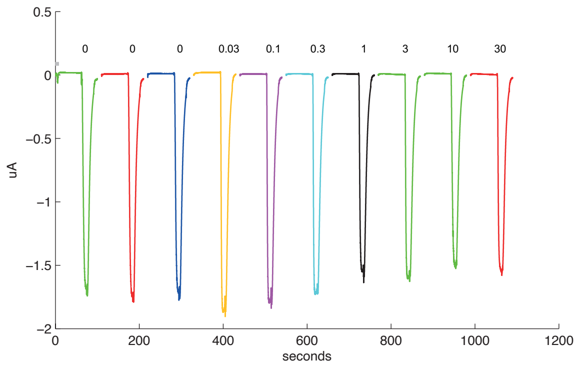

Clu-GABA α5β2γ2

Supplementary Figure 3. Current traces from a co-application of GABA and increasing dosage of the compound (in µM) form respectively six and five different *Homo sapiens sapiens* and *Canis lupus familiaris* GABA receptor subtypes.
