# Supplementary material for: Comparative analysis of isoxazoline activity on human and canine GABA receptors expressed in Xenopus oocytes
Source: Parasit Vectors. 2025 Jun 6;18:213. doi: 10.1186/s13071-025-06847-3 (PMC12144822; doi:10.1186/s13071-025-06847-3)
Supplement: Supplementary file 6 — Additional file 6: Table S1. Primers for full-length ORF amplification. [file 13071_2025_6847_MOESM6_ESM.docx]

**Supplementary Table S1**

| **Primer name** | **Sequence 5’ 🡪 3’** |
| --- | --- |
| *Primers for full-length ORF amplification* | |
| BglII_Clu-GABA-a2_F | GGCGAGATCTCCCCCTTGCATGGCTTTG |
| XhoI_Clu-GABA-a2_R | GGCGCTCGAGTTCAAGGGCTGACCCCTAAT |
| NheI_Clu-GABA-a3_F | GGCGGCTAGCCACACAAATGAGTCACTTCTACACA |
| XhoI_Clu-GABA-a3_R | GGCGCTCGAGCTAGTGTTTGCGGATCATGC |
| NheI_Clu-GABA-a5_F | GGCGGCTAGCATTGGGAATGGACAATGGAA |
| XhoI_Clu-GABA-a5_R | GGCGCTCGAGGCTCTCTTGGAGTTTGGGAGT |
| NheI_Clu-GABA-b3_F | GGCGGCTAGCGAGGGATGTGGGGCTTTG |
| XhoI_Clu-GABA-b3_R | GGCGCTCGAGTCACTCAGTTAACATAGTACAGCCAGT |
